# HPLC Estimation of berberine in *Tinospora cordifolia* and *Tinospora sinensis*

**DOI:** 10.4103/0250-474X.40341

**Published:** 2008

**Authors:** G. V. Srinivasan, K. P. Unnikrishnan, A. B. Rema Shree, Indira Balachandran

**Affiliations:** Phytochemistry Division, Centre for Medicinal Plants Research, Arya Vaidya Sala, Changuvetty, Kottakkal - 676 503, Kerala, India

**Keywords:** *Tinospora cordifolia*, *Tinospora sinensis*, berberine, HPLC

## Abstract

A high-performance liquid chromatographic method for the estimation of berberine in the stem of *Tinospora cordifolia* (Willd.) Miers. ex Hook.f. and Thoms. and *Tinospora sinensis* (Lour.) Merrill is described. The dried stems of *T. cordifolia* and *T. sinensis* were defatted with petroleum ether (60-80°). The marc was dried and further extracted with methanol. The concentration of berberine in methanol extract was determined using a C-18 reverse phase column with a mobile phase of acetonitrile:water (10:90 v/v) at a flow rate of 0.6 ml/min and with UV detection at 266 nm. TLC and HPLC comparison of both the species revealed significant variation in the chemical constitution of the two species. This observation becomes important in the context of the use of *T. sinensis* in place of the genuine drug *T. cordifolia*.

*T. cordifolia* (Family: Menispermaceae), known as *Amrita* (*Guduchi*) in Sanskrit is a widely used plant in folk and Ayurvedic systems of medicine. The term *Amrita* meaning divine nectar is attributed to this drug in recognition of its capacity to impart youthfulness, vitality, and longevity to the consumer^1^. Drug consists of the dried stem with bark intact. It is widely used in folk and Ayurvedic systems of medicine for its general tonic, anticancer^2^, antiulcer^3^, antipyretic^4^, antihepatitis^5^, immunomodulatory^6^, antioxidant^7^, hypoglycaemic^8^, antineoplastic, cardiotonic, antibacterial, antimicrobial, antileishmanial, antiinflammatory, antiarthritic, analgesic and diuretic^9^,^14^ properties. The drug is reported to possess 20% of the analgesic effect of sodium salicylate^10^,^12^. The plant is used in Ayurvedic *Rasayanas* to improve the immune system and the body resistance against infections^11^. *Amrita* is a constituent of several preparations like *Amritarishtam*, *Dhanvantaram tailam*, *Cheriya rasnadi kashayam* and *Valiya marmagulika*^1^. *T. sinensis* (Fam: Menispermaceae) is used almost in the same way as *T. cordifolia*^12^. However, practitioners consider *T. cordifolia* as the genuine source for *Amrita.*

Sesquiterpene tinocordifolin^15^, sesquiterpene glycoside tinocordifolioside^16^, an immunologically active arabinogalactan^17^, phytoecdysones^18^ viz., ecdysterone and makisterone, Alkaloids^19^,^20^ viz., berberine and magnoflorine are the major chemical compounds isolated from the stem of *T. cordifolia*. Magnoflorine, berberine, tinosporicide, menispermacide, palmatine, (+)− malabarolide and tinosinen I are the major chemical compounds isolated from the stem of *T. sinensis*^21^–^23^.

A reverse phase HPLC method has been developed to quantitatively estimate the berberine content in the stem of *T. cordifolia* and *T. sinensis*. Berberine (B_1_) is an isoquinoline alkaloid reported to have antimalarial, antipyretic, antimicrobial, antibacterial, antitumour and antiprotozoal (*Leishmania*) activities^13^,^14^. An attempt has also been made to chromatographically compare the methanolic extracts of both the species.

The stem of *T. cordifolia* and *T. sinensis* were collected from the Herb Garden, Arya Vaidya Sala, Kottakkal, Kerala State, India in July 2006 and voucher specimens were deposited at the Herbarium, Centre for Medicinal Plants Research, Kottakkal, Kerala (Voucher No. 02202, 01363, 02426, and 03378). The stem was dried in the shade and coarsely powdered. Powdered material of *T. cordifolia* and *T. sinensis* (5 g) were defatted with 100 ml petroleum ether in a Soxhlet extractor for 12 h. The marc was air-dried and was further extracted with 50 ml methanol in a Soxhlet extractor for 12 h at 60°. The extract was filtered and concentrated to dryness under reduced pressure below 60° using a rotary flash evaporator. Different concentrations of the residue were prepared in methanol and used for HPLC analysis to quantify the berberine content.

Solvents used were of HPLC grade (E. Merck). Test solutions were filtered through 0.20 μm nylon-6,6 membrane before injection. All analyses were run in triplicate and averaged. The standard berberine used was purchased from Fluka Chemicals, Switzerland. TLC was done on pre- coated silica gel 60 F_254_ plates (E. Merck) of uniform thickness of 0.2 mm.

A Shimadzu (Kyoto, Japan) HPLC system consisted of LC-10AT VP pump, SPD-M10A VP photodiode array detector, CLASS-VP 6.12 SP5 integration software and a Rheodyne injection valve fitted with a 20 μl injection loop, was used for the analysis. Baseline resolution of B_1_ was obtained at 25 ± 2° using a Phenomenex Luna C-18 column (250 × 4.6 mm i.d; 5 μm) and an isocratic solvent system consisting of acetonitrile-water in the ratio 10:90 (v/v). The mobile phase was passed through 0.45 μm PVDF filter, degassed before use. The flow rate was kept constant at 0.6 ml/min and the detection was at 266 nm. For calibration, standard solutions of B_1_ were prepared at concentrations of 1, 5, 10, 20, 40, 50, 80, 100, 200, 400, 800 and 1000 μg/ml using methanol as solvent. The standard solution was injected in triplicate and the average detector response was measured. The stem extracts were assayed in triplicate and peak areas corresponding to B_1_ were compared with the calibration curve and amount of B_1_ was determined.

The validated parameters were specificity, linearity, precision and accuracy according to the ICH guidelines^24^. Inter-day reproducibility was verified by analyzing six different concentrations of B_1_, each injected four times, and determining the relative standard deviation (RSD%). The intra-day reproducibility was evaluated by the analysis of B_1_ at two different concentrations (80 and 100 μg/ml), four times a day on seven consecutive days and determining the RSD%. For recovery studies, 500 μl of methanol solution containing 100 μg ml of B_1_ was added to 500 μl of three methanol extract solutions (10, 20, 40 mg/ml). Recovery was calculated by comparing the resulting peak area with the peak obtained from equal concentrations of B_1_ and expressed as percentage of this ratio.

Thin layer and HPLC analyses were carried out to compare the crude methanolic extracts of *T. cordifolia* and *T. sinensis.* Thin layer chromatography was performed on precoated silica gel 60 F_254_ TLC plates (E. Merck) of uniform thickness of 0.2 mm. The chromatogram was developed up to 80 mm under chamber saturation conditions with chloroform-methanol, 70:20 (v/v) in a twin trough chamber. For HPLC analysis the conditions mentioned above were maintained.

In view of the potential therapeutic importance of the drug *Amrita*, and considering the degree of adulteration/substitution of the raw materials, a simple HPLC method was developed and validated in order to quantify berberine (B_1_). Satisfactory retention times and good resolution of B_1_ was achieved using reverse phase C-18 column eluted with acetonitrile-water (10:90 v/v) at a flow rate of 0.6 ml/min. A sharp and symmetric peak for B_1_ was obtained, with good baseline resolution and minimal tailing, thus facilitating the accurate measurement of peak area. The HPLC analysis was carried out in isocratic conditions and a retention time of 8.65 min was obtained for standard berberine. Typical HPLC chromatograms of B_1_ and methanol extracts of the stem of *T. cordifolia* and *T. sinensis* are shown in figs. 1–3.

**Fig. 1:**
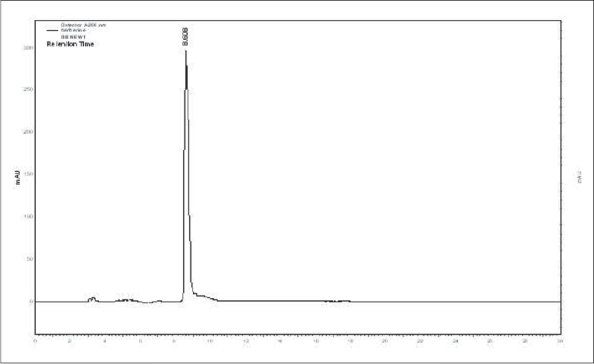
HPLC chromatogram of standard berberine. Berberine peak at the retetion time 8.6 min detected at a wavelength of 266 nm.

**Fig. 2:**
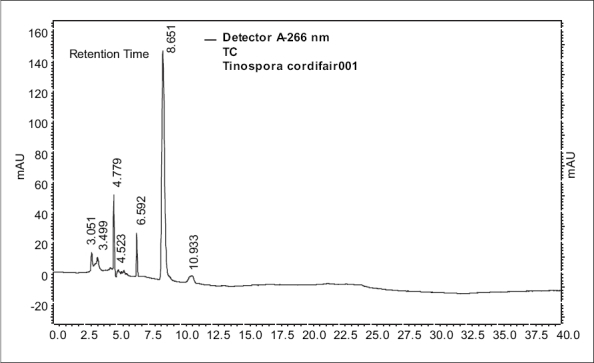
HPLC chromatogram of methanol extract of *T. cordifolia*. Peak at the retention time 8.6 min correspond to berberine.

**Fig. 3:**
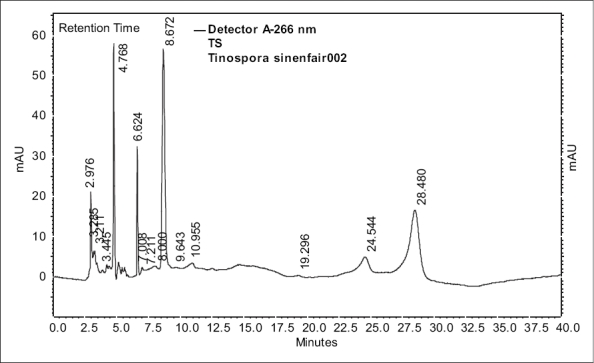
HPLC chromatogram of methanol extract of *T. sinensis*. Peak at the retention time 8.6 min correspond to berberine.

The calibration curve for B_1_ was found to be linear over the range 0.1 to 0.01 mg/ml (r^2^ = 0.98). The linear regression equation for the calibration curve was y = 123512x -78787, where y is the peak area ratio of B_1_ and x is the concentration of B_1_ (μg/ml). The average recovery was 99.10±0.32%, while the inter-day and intra-day reproducibility were found to be 3.5% and 4.7%, respectively. The concentration of B_1_ in the stem of *T. cordifolia* and *T. sinensis* (dry weight basis) was found to be 0.3192% and 0.0967% (w/w), respectively (Table 1).

**TABLE 1:** CONCENTRATION OF BERBERINE IN DIFFERENT SAMPLES

Plant drug	Concentration of berberine (%) in the samples^a^			

	Sample 1	Sample 2	Sample 3	Average Conc. (%)^b^
*T. cordifolia*	0.3196	0.3188	0.3191	0.3192
*T. sinensis*	0.0965	0.0971	0.0966	0.0967

a Methanol extract of the defatted plant drug

b concentration of berberine in the dried plant drugs

Comparative chromatographic studies of the methanol extract of the two species revealed the presence of two additional compounds in *T. sinensis*. These two compounds were isolated by column chromatography followed by preparative TLC. HPLC analysis gave peaks at 24.54 and 28.48 min, in the methanol extract of *T. sinensis*. Preliminary studies (UV, FTIR, TLC and derivatisation) indicate that these two compounds possess a steroid nucleus. Detailed characterization studies of these compounds are under progress.

Ayurvedic practitioners of Kerala use both *T. coridifolia* and *T. sinensis* as *Amrita*. Present study revealed that *T. cordifolia* and *T. sinensis* show differences in chemical constituents. The berberine content of the two species show marked variation, *T. cordifolia* having three times higher berberine concentration. On the other hand *T. sinensis* contain certain compounds that do not occur in *T. cordifolia.* Whether they are of any therapeutic significance is not known and hence need to be investigated. Such chemical variation highlights the need for pharmacological standardization and validation. The analytical conditions presented here are applicable with respect to the identification and quantification of B_1_ and for the quality checking of raw drugs to distinguish the two species conveniently.
